# Predictors of nonresponse and drop-out among children and adolescents receiving TF-CBT: investigation of client-, therapist-, and implementation factors

**DOI:** 10.1186/s12913-022-08497-y

**Published:** 2022-09-29

**Authors:** Ane-Marthe Solheim Skar, Nora Braathu, Tine K. Jensen, Silje Mørup Ormhaug

**Affiliations:** 1 Norwegian Center for Violence and Traumatic Stress Studies, Gullhaugveien 1-3, 0484 Oslo, Norway; 2 Department of Psychology, University of Oslo, Forskningsveien 3A, Oslo, Norway

**Keywords:** Attrition, Evidence-based practice, Treatment interruption

## Abstract

**Background:**

There is a paucity of evidence about effective implementation strategies to increase treatment response and prevent drop-out among children receiving evidence-based treatment. This study examines patient, therapist, and implementation factors and their association to nonresponse and drop-out among youth receiving Trauma-Focused Cognitive Behavioral Therapy (TF-CBT).

**Methods:**

Youth (*n* = 1240) aged 6–18 (M = 14.6) received TF-CBT delivered by 382 TF-CBT therapists at 66 clinics. Odds ratio analyses were used to investigate whether pretreatment child (age, gender, number of trauma experiences, post-traumatic stress symptoms (PTSS), therapist (education), and implementation strategy factors (high-low, low-low, low–high intensity therapist and leadership training respectively) or tele-mental health training during the Covid-19 pandemic are associated with nonresponse (above clinical PTSS level post-treatment) and drop-out (therapist-defined early termination). Fidelity checks were conducted to ensure that TF-CBT was used consistently.

**Results:**

One fourth of the children (24.4%) were nonresponders and 13.3 percent dropped out. Exposure to three or more traumatic experiences were related to nonresponse and drop-out. Higher baseline PTSS was related to a higher probability of nonresponse. There was no effect of therapist education or child gender on nonresponse and drop-out, whereas children over 15 years had a higher likelihood of both. After controlling for baseline PTSS, the effect of age on nonresponse was no longer significant. Drop-out was related to fewer sessions, and most dropped out during the first two phases of TF-CBT. Fidelity was high throughout the different implementation phases. High-intensity therapist training was related to a lower probability of both nonresponse and drop-out, whereas low therapist and leadership training were related to a higher likelihood of both. Multivariate analysis revealed higher child age and higher PTSS baseline scores as significant predictors of nonresponse, and number of trauma experiences (> = 3) at baseline as the only predictor of drop-out.

**Conclusions:**

High-intensity therapist training seem key to prevent patient nonresponse and drop-out. Leadership training might positively affect both, although not enough to compensate for less intensive therapist training. More complex cases (higher PTSS and exposure to more traumas) predict nonresponse and drop-out respectively, which underscores the importance of symptom assessment to tailor the treatment. The lack of predictive effect of therapist education increases the utilization of TF-CBT.

**Trial registration:**

Retrospectively registered in ClinicalTrials, ref. nr. NCT05248971.

## Introduction

Despite the effectiveness of a variety of Evidence-Based Practices (EBPs) for mental health problems among children and adolescents [8, 61], there is a concern that some children drop out from treatment or do not respond sufficiently, with the likely consequence that their problems will worsen or become chronic. Trauma is a critical factor for mental health problems, and research has shown that exposure to childhood trauma is accountable for approximately 45 percent of all childhood-onset disorders [25, 55] and approximately one third of all onsets of mental health disorders across the globe [25, 34, 39]. Approximately 16 percent of those exposed to childhood trauma develop post-traumatic stress disorder (PTSD) [4] and even more experience subclinical levels of post-traumatic stress symptoms (PTSS). Despite the proven effectiveness of trauma focused EBPs for PTSS as compared to therapy as usual among youth [36, 38], review and meta-analytic studies find nonresponse rates up to 50 percent [33] and an average drop-out rate of 11.7 percent across treatment methods [52].

Trauma-Focused Cognitive Behavioral Therapy (TF-CBT) is considered the treatment of choice for youth who have developed elevated PTSS [29, 42]. Nonetheless, a substantial portion of the patients receiving TF-CBT are treatment non-responders. In a study from Norwegian child and adolescent mental health services, 18.2 percent fulfilled the diagnostic criteria for PTSD following treatment with TF-CBT (36.1% in the therapy as usual condition) [31]. Previous research has investigated individual and setting barriers related to nonresponse in TF-CBT and identified female gender [35] and higher pretreatment PTSS [35] as significant predictors of nonresponse. One study found an association between greater response from TF-CBT and higher treatment fidelity [5]. These studies imply that both child-related factors and treatment-related factors are related to treatment success.

Another challenge for treatment uptake is premature drop-out. A meta-analysis including 40 studies addressing drop-out from EBPs for PTSD in children and adolescents found an average drop-out rate of 11.2 percent for TF-CBT; similar to the rates for non-trauma treatments and wait-list conditions [52]. Yet individual studies find a range in drop-out level from 11 [11] to 27 [30, 66] percent, which might be explained by the variation in how drop-out has been defined in the various studies, i.e., completion of fewer than a certain number of therapy sessions (dose definition) [11, 12, 17, 19, 30, 31, 66], or based on clinical judgment (e.g., [44]).

Several studies have aimed to identify predictors of drop-out. In one meta-analysis, neither type of trauma (single versus multiple), type of EBT for PTSD, or number of treatment sessions had any significant impact on drop-out [52]. On the other hand, individual studies on TF-CBT have found that older age and being exposed to a higher number of different traumatic events [31], higher caregiver-rated pretreatment symptoms [59], being in foster-care [66], in-session child and caregiver avoidance [66], and therapeutic relationship problems [44, 66] are related to drop-out. Therefore, focus on both individual and system-level factors are warranted to broaden our understanding about drop-out.

An area of research that so far has been neglected is the relationship between nonresponse and drop-out, and implementation strategies, that is”methods or techniques used to enhance the adoption, implementation, and sustainability of a clinical program or practice” [15]. Implementation research has taught us that the success of EBP implementation depends not only on *what* is being implemented, but also *how* it is implemented, hence knowledge on the effect of different implementation strategies on patient outcomes might inform evidence-supported implementation strategies to increase treatment uptake and prevent drop-out. A synthesis of the implementation literature identified 73 different implementation strategies, of whom training and consultations are among the most common [46]. Yet, little is known about the association between training and consultation dosage and nonresponse and drop-out. Also, to be able to develop preventive strategies to minimize drop-out, there is a need to understand when in the therapeutic process drop-out is most likely to happen. Implementation leadership has demonstrated to play a key role to succeed with implementation efforts (e.g., [1]). Theoretical models have suggested that implementation leadership enables a supportive implementation climate, which again is linked to positive attitudes to EBPs and higher fidelity to the treatment model among practitioners, likely having a positive impact on patient outcomes [21]. Studies investigating the association between implementation leadership training and patient nonresponse and drop-out would add valuable information that could guide the implementation of EBPs. Lastly, during the Covid-19 pandemic, the world had to quickly develop and implement new routines and ways of working. Within the health sector, this often included using digital tools for training and consultations of EBPs. As digital solutions are likely to play a larger part in the society than previously also post Covid, there is a need to understand the implications of moving the traditional face-to-face courses in EBPs into the virtual meeting room.

Against this background, the current study examines child and therapist factors related to nonresponse and drop-out, as well as the associations between implementation factors and nonresponse and drop-out from TF-CBT, thus expanding the current state of knowledge. More specifically, the present study aims to investigate the predictive effect of *patient factors* (pretreatment age, gender, number of trauma experiences, and level of PTSS), *therapist* (educational background), and *implementation strategy* (training and consultation dosages and leadership training) factors on nonresponse and drop-out. In addition, we aim to investigate the effect of digital training and consultation in TF-CBT which was sparked due to the Covid-19 pandemic. Lastly, we will investigate when drop-out occurs across the different phases in TF-CBT. To ensure that the therapy provided were in line with the TF-CBT protocol, fidelity scores will be investigated. Knowledge about individual patient and therapist factors as well as system factors related to nonresponse and drop-out is vital for treatment planning at both the individual and system level to ensure that as many as possible benefit from EBPs.

## Methods

### Participants

The patient sample comprises 1240 youth receiving TF-CBT in child and adolescent mental health services. A total of 793 (64%) were girls and 274 (22.1%) were boys (*n* = 173 [14%] missing). They were aged 6–18, with a mean age of 14.6 (SD = 2.75) at intake (*n* = 211 [17%] missing). Girls had a higher mean age (15.1, SD = 2.48) than boys (13.4, SD = 2.99) (t = -8.26, *p* < 0.001). Further, girls had a higher level of PTSS symptoms at baseline (M = 34.8, SD = 9.74) compared to boys (M = 29.9, SD = 9.11) (t = -7.53, p = 001). The patients reported exposure to a mean number of 3.2 different potential traumatizing experiences, with severe bullying or threats being the most frequent, reported by 34.9 percent of the participants (see Table 1).

**Table 1:** Number and type of trauma experiences, PTSS at baseline, and change in PTSS from pre- to post-treatment among youth receiving TF-CBT

	Overall^a^ (*N* = 1240)	Girls (*N* = 793)	Boys (*N* = 274)	Test of difference^b^ t-test/ exact chi square *p*-value	Cramer’s V / Cohen’s D^c^
**Number of traumas**					
Mean (SD)	3.24 (1.41)	3.30 (1.46)	3.04 (1.27)	.459	-0.05 (-0.19 – 0.09)
Median [Min, Max]	3 [1.0, 6.0]	3 [1.0, 6.0]	3 [1.0, 6.0]		
**Sexual Abuse**	391 (31.5%)	349 (44.0%)	39 (14.2%)	< .001	0.28 (0.23–0.31)
**Community Violence**	362 (29.2%)	252 (31.8%)	107 (39.1%)	.035	0.07 (0.01–0.13)
**Severe bullying or threats**	433 (34.9%)	321 (40.5%)	109 (39.8%)	.742	0.01 (0.00–0.07)
**Domestic Violence**	383 (30.9%)	275 (34.7%)	107 (39.1%)	.230	0.04 (0.00–0.10)
**Accidents/Illness**	420 (33.9%)	309 (39.0%)	108 (39.4%)	.990	0.00 (0.00–0.07)
**PTSS baseline**					
Mean (SD)	33.5 (9.74)	34.8 (9.74)	29.9 (9.11)	< .001	-0.51 (-0.65—-0.37)
Median [Min, Max]	33.0 [10.0, 67.0]	35.0 [10.0, 67.0]	29.0 [15.0, 56.0]		
**PTSS change (pre-post)**					
Mean (SD)	18.6 (11.6)	18.9 (11.4)	16.1 (11.9)	.010	-0.24 (-0.42—-0.06)
Median [Min, Max]	19.0 [-31.0, 52.0]	19.0 [-13.0, 52.0]	16.0 [-31.0, 50.0]		
Missing	520 (41.9%)	354 (44.6%)	105 (38.3%)		

^a^Due to missing data on gender, the overall mean differs from the mean for boys and girls, respectively

^b^Tests refer to differences between the boys and girls

^c^Cramer’s V estimate was used for categorical variables, Cohen’s D was used for continuous scores

The practitioner sample consists of 382 TF-CBT therapists from 66 child and adolescent mental health services. The educational background of the therapist spanned from clinical psychologist (*n*=176, 46.1%), 3-year health education with 2 years of clinical specialization (*n*=72, 18.8%), 3-year health education (*n*=29, 7.6%), medical doctor or psychiatrist (*n*=22, 5.8%) (*n*=83 [21.7%] missing).

### Trauma-Focused Cognitive Behavioral Therapy (TF-CBT)

Trauma-Focused Cognitive Behavioral Therapy (TF-CBT) [13, 14] is a phase- and component-based treatment method for youth with clinical elevated PTSS following trauma exposure. The components are summarized as the acronym P.R.A.C.T.I.C.E – Psychoeducation and parenting skills, Relaxation skills, Affective and modulation skills, and Cognitive coping skills (the *skills phase*); Trauma narrative and cognitive processing of the traumatic event(s) (the *narrative processing phase*); and In vivo mastery of trauma reminders, Conjoint caregiver-child sessions, and Enhancing future safety and positive development (the *consolidating phase*). Non-offending caregivers are included in parallel sessions with the child. TF-CBT is delivered by trained TF-CBT therapists, normally through 12–15 therapy sessions, yet the number of sessions is adjusted to meet the needs of the individual child and family.

### Setting

The study was conducted in child and adolescent mental health clinics located across the five health trusts in Norway. All clinics were part of a nationwide state-funded TF-CBT implementation covering 75 percent of all clinics. Children are referred to the specialist services by their medical doctor, the child protective services, or municipality first-line services.

### Procedures

Data were collected in 2015–2021. At a clinic level, inclusion criteria were that the leadership would provide time for the therapists to learn and use TF-CBT, that the clinic would implement systems for routine trauma screening, and that the leaders would take part in implementation leadership activities to support the implementation of TF-CBT. Inclusion criteria at a therapist level included working as a therapist in their current position and being motivated to learn TF-CBT. These criteria were included in the collaborating agreement signed by each clinic and the implementing institution. Inclusion criteria for patients receiving TF-CBT were age between 6–18 and clinically significant PTSS (≥ 15) based on the Child and Adolescent Trauma Screen (CATS; [50, 51].

#### Implementation strategies

**Fig. 1:**
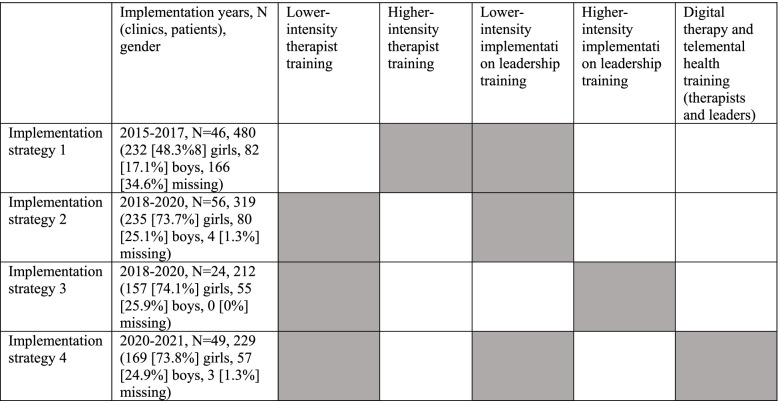
Implementation strategies

Different single implementation strategies were used simultaneously, i.e. a multifaceted approach [26], for therapist and implementation leadership training and subsequent consultations, with higher and lower intensity. The implementation strategies were selected and tailored based on identified determinants (facilitators and barriers). The implementation strategies used (in italic) are named in accordance with the Expert Recommendations for Implementing Change (ERIC) [46]. Implementation strategy 1 included higher-intensity therapist training and lower-intensity leadership training. Implementation strategy 2 included lower-intensity therapist training and lower-intensity leadership training. Implementation strategy 3 included lower-intensity therapist training and higher-intensity leadership training. Finally, implementation strategy 4 included lower-intensity therapist training and lower-intensity leadership training, and the therapist and leadership training were fully digital while the therapy sessions with the patients were partly digital (see Fig. 1).

Lower-intensity therapist training included the following:

- *Educational meetings*: All therapists at the clinic received in-person training in trauma and PTSS screening and assessment. A sub-group of therapists received training in TF-CBT and participated in a 1-day pre-recorded digital course followed by a 2-day in-person training course in TF-CBT led by TF-CBT trainers.
- *Audit and provide feedback*: The TF-CBT trainers listened to the audio recording of an average of one case based on clinical judgement of the therapists’ need.
- *Provide ongoing consultations and clinical supervision*: Weekly general case consultations with groups of 4–5 therapists. Therapists could present cases and challenges, which were discussed in the group. The trainers did not systematically listen to audio recordings of ongoing therapies before the consultations.

Higher-intensity therapist training included the following:

- *Educational meetings*: All therapists at the clinic received in-person training in trauma and PTSS screening and assessment. A sub-group of therapists received training in TF-CBT and participated in a 3-day in-person training course in TF-CBT led by TF-CBT trainers.
- *Audit and provide feedback*: The TF-CBT trainers listened to the audio recording of all sessions in the therapists' three first cases and provided feedback.
- *Provide ongoing consultations and clinical supervision*: Weekly session-by-session case consultations with groups of 4–5 therapists. The trainers systematically listened to audio recordings of ongoing therapies before the consultations which formed the basis for the consultations. In addition, the therapists could present challenges, which were discussed in the group.

Lower-intensity implementation leadership training included the following:

- *Recruit, designate, and train for leadership* and *Tailor the strategies and purposely reexamine the implementation*: Monthly 30–60 min semi-structured consultation calls with the clinic leaders focusing on troubleshooting.

Higher-intensity implementation leadership training included the following:

- *Recruit, designate, and train for leadership* and *Tailor the strategies and purposely reexamine the implementation*: Leaders received high-intensity training through participating in the one-year Leadership and Organizational Change for Implementation (LOCI) to support and strengthen implementation leadership and implementation climate [1]. This included a 360 degrees assessment of implementation and general leadership skills among first-level leaders and implementation climate at the clinic level every fourth month for a year, a 2-days in-person workshop at baseline and a 1-day in-person workshop at month 4, 8, and 12; leadership development plans based on individual feedback reports from the 360-assessments; organizational implementation plans based on aggregated data on implementation climate from the 360-assessments; weekly consultation calls (30 min) with first-level leaders and monthly strategic meetings (30-60 min) with the executive management to follow up on the leadership and organizational development plans respectively.

### Measures

The therapists provided data on the child's age, gender, trauma exposure, and symptoms of posttraumatic stress at baseline and post-treatment. Gender was coded as 0 = boys and 1 = girls.

#### Potentially traumatizing events

A 15-item self-report screening developed for use within mental health services in Norway for children between 6 and 18 years was used to measure exposure to potentially traumatizing events according to the DSM-5 definition [6]. Respondents answered *yes* or *no* to the different potentially traumatizing events (sexual abuse, community violence, severe bullying or threats, domestic violence, accidents/illness). The number of different events experienced by the child is added to create a total trauma score.

#### Posttraumatic stress symptoms

Posttraumatic stress symptoms were measured using the Child and Adolescent Trauma Screen (CATS) [50]. From 2018, version 2.0 of CATS was used [51]. The CATS is based on the diagnostic criteria in the Diagnostic and Statistical Manual of Mental Disorders, Fifth Edition (DSM-5), and version 2.0 measures PTSS according to both DSM 5 and ICD-11. Scores are added for a total symptom severity score (ranging from 0 to 60). In the current dataset, we only had access to sum scores of the measure, and we could therefore not examine its internal reliability. However, CATS has been widely used internationally with good psychometric properties [50, 51]. Recommended clinical cut-off score for probable PTSD is ≥ 21 points.

#### Fidelity

An online quality monitoring system was developed for therapy self-monitoring of treatment sessions. Furthermore, TF-CBT trainers scored treatment fidelity using a modified version of the TF-CBT Brief Practice Fidelity Checklist [16] based on audio recordings of treatment sessions. Fidelity was coded as 0 = fidelity is not met, and 1 = fidelity is met.

#### Nonresponse and drop-out

Nonresponse was conceptualized as posttraumatic stress symptoms above the clinical cut-off for probable PTSD (≥ 21) after receiving the full TF-CBT protocol (patients who scored below the clinical cut-off for possible PTSD at baseline were excluded from this analysis). Treatment drop-out was conceptualized as not receiving the full TF-CBT protocol according to therapist judgement, and coded as 0 = no retention, and 1 = retention. This means that therapists recorded drop-out in cases when the patients stopped showing up at the therapy sessions when this was not agreed upon between the therapist and the patient.

### Analysis

The analyses were done in R studio [48]. Odds ratio was used to investigate the relationship between the dependent variables (drop-out and nonresponse) and the individual patient, therapist, therapy, and implementation factors. The odds ratio estimate was exponentiated after running a generalized linear mixed effect model using the glmer function in the lme4 package in R [7]. To account for the nested data, we included clinic as a random effect. Odds ratio signifies the odds that an outcome will occur in one group, compared to the odds of the outcome occurring in another group. OR = 1 means that exposure does not affect odds of outcome, OR > 1 that exposure is associated with higher odds of outcome; and OR < 1 that exposure is associated with lower odds of outcome [54]. All variables were first analyzed individually, and then with the adjustment of PTSS scores at baseline.

T-tests and chi-square analyses were reported to illustrate differences between girls and boys regarding number and type of trauma experiences, PTSS at baseline and change in PTSS from pre- to post-treatment. Trauma experiences was coded as 0 = under 3, and 1 = 3 or above. This cut-off was used as it was the median number. To investigate differences between the four implementation strategies and whether the sessions had adequate fidelity or not, we used the “glmer” function in the R package “lme4” [7]. This function allows for fixed effects parameters and random effects. In the current case, we used patients as random effects. We used Cramer’s V to report effect sizes between two categorical variables, for example between drop-out and gender [3], and Cohen’s D with at least one continuous variable, for example between PTSS baseline scores and gender.

## Results

Patients (*n* = 1240) had a mean PTSS score of 33.5 (SD = 9.74) pretreatment, and 14.1 (*SD* = 10.6) at the end of treatment (t = -7.53, *p* < 0.001). When controlling for baseline PTSS scores, girls had a significantly larger drop in PTSS scores (M = 18.9-point drop on the CATS) from baseline to end of treatment compared to boys (M = 16.1-point drop) (t = -2.61, *p* = 0.001) (see Table 1).

### Fidelity

Fidelity was high throughout the different implementation phases. In implementation strategy 1, 92.4 percent (*n* = 6601) of the 7142 sessions scored met the criteria for fidelity [16]. In strategy 2, the percentage was 87.5 (553 of 632 sessions scored), in strategy 3 the fidelity score was 91.0 (559 of 614 sessions scored), and in strategy 4 the percentage was 91.7 (1144 of 1248 sessions scored). Generalized mixed effects analyses showed that implementation strategy 1 had a significantly higher number of sessions with fidelity when compared to implementation strategy 2 (Estimate = 0.49, *p* = 0.027). Implementation strategies 3 and 4 did not significantly differ from implementation strategy 1 (*p* = 0.689, *p* = 0.683), or when using any of the other strategies as reference. As the number of participants in each strategy varies, we conducted a chi-square test to examine differences between the two variables. The results show a significant difference between the implementation strategies and fidelity (Estimate = 19.8, *p* < 0.001). However, the chi-square estimate does not account for nested data. The Cramer’s V estimate was 0.05 (CI = 0.03 – 0.07). Both the generalized mixed effects analysis and the chi-square test indicate significant differences between the implementation strategies and fidelity.

### Nonresponse

Almost one fourth of the patients (24.4%) who completed TF-CBT were categorized as non-responders, as they scored above the clinical cut-off for possible PTSD (≥ 21) at the end of treatment. The bivariate relationship between nonresponse and patient, therapist, and implementation factors are listed in Table 2. Patients under 15 years old were less likely to not respond to therapy compared to older participants (OR = 0.64, *p* = 0.039). However, after controlling for PTSS baseline scores, age was no longer significantly related to nonresponse (OR = 0.85, *p* = 0.468). There was no significant difference in nonresponse based on child gender. Patients with PTSS scores above 33 (median PTSS score) pretreatment (OR = 3.05, *p* < 0.001) and patients who reported three or more potential traumatizing experiences (OR = 1.90, *p* < 0.001) were more likely to be categorized as nonresponders.

**Table 2:** Bivariate analyses. Nonresponse and patient, therapist, and implementation factors

**Variable**	DF*	***p*-value**	**Odds ratio**	**Confidence intervals** Lower CI Upper CI		**Cramer’s V estimate** (Lower CI – Upper CI)
**Patient factors**						
Age (< = 15)	516	.039	0.64	0.42	0.97	0.09 (0.01–0.17)
Gender (girl)	545	.142	1.39	0.90	2.19	0.06 (0.00–0.14)
> = 3 trauma experiences	644	< .001	1.90	1.32	2.73	0.13 (0.05–0.21)
PTSS pretreatment (total score > 33)	644	< .001	3.05	2.09	4.53	0.23 (0.16–0.31)
**Therapist factor (educational background)**						
Bachelor	514	.304	1.45	0.69	2.91	0.06 (0.00–0.14)
Master	514	.974	1.27	0.77	2.05	0.05 (0.00–0.15)
Medicine	514	.196	0.64	0.37	1.10	0.07 (0.01–0.15)
Psychologist	514	.614	0.88	0.57	1.35	0.02 (0.00–0.11)
**Implementation factor**						
Implementation strategy 1	644	< .001	0.50	0.34	0.73	0.16 (0–08-0.24)
Implementation strategy 2	644	.010	1.74	1.13	2.65	0.11 (0.04–0.19)
Implementation strategy 3	644	.420	1.22	0.74	1.95	0.05 (0.00–0.13)
Implementation strategy 4	644	.356	1.32	0.72	2.37	0.05 (0.00–0.14)

^*^Sample size is indicated by the degrees of freedom (DF) + 1. The sample size for each parameter varies due to missing values in the data set

There was no significant effect of the educational background of the therapists on nonresponse. Regarding the implementation strategy, results showed that patients from clinics receiving implementation strategy 1 were less likely to not respond compared to clinics receiving implementation strategies 2–4 (OR = 0.50, *p* < 0.001). Those receiving implementation strategy 2 were more likely to not respond compared to the three other strategies (OR = 1.74, *p* = 0.010). No other variable than "Age" changed significantly after adjusting for PTSS scores at baseline, which changed to not being significant. When adding all variables in a multivariate model, age (< 15, OR = 0.59, *p* = 0.048) and higher PTSS levels at baseline (OR = 2.80, *p* =  < 0.001) were significant predictors of nonresponse. As seen in Table 2, the effect size estimates were relatively low for all variables.

### Drop-out

A total of 13.3 percent of the patients dropped out from the treatment. Table 3 summarizes the bivariate analysis results investigating the ratio between drop-out and patient, therapist, and implementation factors. Firstly, there was a statistically significant difference between patients under and over the age of 15, with the former being less likely to drop out (OR = 0.65, *p* = 0.016). There was no significant effect of child gender on drop-out (OR = 1.25, *p* = 0.271). Patients reporting exposure to three or more potentially traumatizing events were more likely to drop out than those who reported fewer than three traumas (OR = 1.74, *p* < 0.001). Patients with PTSS pretreatment scores over 33 (median PTSS score) did not differ significantly from those under 33 on the likelihood of drop-out (OR = 1.32, *p* = 0.104). Among those dropping out, the majority did so during the Skills phase (40.0%), followed by the Narrative phase (38.8) and the Consolidation phase (10.9%). There was no significant difference between the educational background of the therapists on patient drop-out.

**Table 3:** Bivariate analyses. Drop-out and patient, therapist, and implementation factors

**Variable**	DF*	***p*-value**	**Odds ratio**	**Confidence intervals** Lower CI Upper CI		**Cramer’s V estimate** **(Lower CI – Upper CI)**
**Patient factors**						
Age (< 15)	1032	.016	0.65	0.44	0.93	0.07 (0.02–0.13)
Gender (girl)	1066	.271	1.25	0.85	1.90	0.03 (0.00–0.09)
> = 3 trauma experiences	751	< .001	1.74	1.17	2.60	0.04 (0.00–0.07)
PTSS pretreatment (total score > 33)	1238	.104	1.32	0.94	1.85	0.05 (0.00–0.10)
**Therapist factor (educational background)**						
Bachelor	951	.722	1.11	0.52	2.13	0.01 (0.00–0.08)
Master	951	.837	1.05	0.65	1.66	0.01 (0.00–0.07)
Medicine	951	.363	0.75	0.38	1.35	0.03 (0.00–0.08)
Psychologist	951	.766	1.06	0.71	1.61	0.01 (0.00–0.08)
**Implementation factor**						
Implementation strategy 1	1238	.008	0.60	0.41	0.87	0.08 (0.03–0.13)
Implementation strategy 2	1238	< .001	2.19	1.54	3.12	0.13 (0.07–0.20)
Implementation strategy 3	1238	.373	0.80	0.49	1.28	0.03 (0.00–0.07)
Implementation strategy 4	1238	.327	0.80	0.50	1.24	0.02 (0.00–0.07)

*Sample size is indicated by the degrees of freedom (DF) + 1. The sample size for each parameter varies due to missing values in the data set

Those in the clinics receiving implementation strategy 1 were less likely to drop out compared to patients in clinics receiving the other implementation strategies (OR = 0.60, *p* = 0.008), and those receiving implementation strategy 2 were more likely to drop out compared to those in clinics who received the three other implementation strategies (OR = 2.19, *p* < 0.001). After controlling for PTSS baseline scores, none of the significant relationships changed. An additional multivariate model with all variables was also computed regarding drop-out. Results revealed that only number of trauma experiences (> = 3) signficantly increased the likelihood of dropping out (OR = 2.41, *p* < 0.001). Similar to the results regarding nonresponse, effect size estimates related to treatment dropout were low (see Table 3).

## Discussion

Nonresponse and drop-out from therapy can be costly for both patients (e.g., continuing suffering), therapists (e.g., feeling of failure and worry about the patient), and healthcare systems (e.g., costs related to mental health issues including health care, drop-out from school, and work absence). To maximize the effect of trauma therapy, there is a need for knowledge on factors related to nonresponse and drop-out to inform the utilization of evidence-supported implementation strategies. This study investigated whether the intensity of training of therapists in TF-CBT and the intensity of leadership training (implementation factor), as well as child age, gender, trauma experiences, and symptoms (child factors), and educational background (therapist factor) predicted nonresponse and drop-out.

Among the 1240 patients, the mean level of PTSS decreased from 33.5 pretreatment to 14.1 post-treatment. Still, about a quarter (24.4%) of those scoring above the clinical level for possible PTSD before treatment also did so following the full TF-CBT treatment protocol. Furthermore, a total of 13.3 percent of the patients dropped out from treatment. This is comparable with results of meta-analysis that found drop-out rates of 11.7 percent from trauma therapy [52] and 14.6 percent for treatment of depression [65]. Yet, a drop-out prevalence of 13.3 percent in the current effectiveness trial is substantially lower than reported in the meta-analysis of de Haan and colleagues [18], which found a drop-out prevalence in trauma treatments of 50 percent in effectiveness studies and 28.4 percent in efficacy studies.

Contrary to Knutsen et al. [35], who found that girls had a higher probability of nonresponse following TF-CBT than boys, there were no differences in nonresponse or drop-out based on gender in the current study. Higher child age (> 15) predicted both nonresponse and drop-out. After controlling for posttraumatic stress scores at baseline, age was no longer a significant predictor of nonresponse. Yet, multivariate analyses revealed both age and higher PTSS baseline scores as significant predictors of nonresponse, hence we might anticipate that both higher age and higher PTSS serves as risks for nonresponse. Knutsen and colleagues [35], investigating responders versus nonresponders among 155 youth receiving TF-CBT, found that baseline PTSS predicted nonresponse. Other studies have demonstrated that lower pretreatment symptoms and fewer trauma experiences predict treatment response among youth receiving TF-CBT [59, 60]. Even though we did not find evidence of any effect on number of trauma experiences on nonresponse, these findings, taken together, indicate that the more complex cases serve as risk factors for treatment outcomes.

In terms of drop-out, adolescents were more likely to terminate the treatment than younger children, even when controlling for level of PTSS pretreatment. This might reflect that adolescents’ developmental trajectories towards independency and a larger focus on peer relations while spending less time with their caregivers might influence their likelihood of making individual choices related to no-shows compared to younger children who are dependent on their caregivers for therapy attendance. Even though we acknowledge that non-significant effects are not indicative for an absent effect in the population [63, 67], the multivariate analysis revealed that number of trauma experiences (> = 3) at baseline was the only predictor of drop-out. Trauma exposure is related to a higher probability of experiencing new traumas (e.g., [23]), and it could be that those experiencing several types of traumas might experience additional mental distress, such as depression or anxiety, which might have influenced drop-out.

The therapists' educational background was not related to the risk of nonresponse or drop-out among the patients, signifying that TF-CBT is a treatment method suitable for clinical therapists with various academic backgrounds. This is in line with findings from Pfeiffer and colleagues [45], who found no association between the educational background of the therapist (*n* = 52) and posttraumatic stress outcomes in the patients (*n* = 153). This is promising, as there is a lack of specialized professionals in many parts of the world. A study from Zambia [41] found decreased trauma and stress-related symptoms following TF-CBT provided by lay health workers, and group formats compared to individual interventions and interventions delivered by lay people compared to professional therapists were related to fewer dropouts in the meta-analysis of Simmons et al. [52]. More research is required on how to effectively implement and scale up EBPs for posttraumatic stress in low-income settings and in low-and-middle income contexts, and the potential of lay health workers and group therapy should be explored further.

Regarding the multifaceted implementation strategies, ranging from low- to high-intensity of therapist and leadership training, patients in the implementation strategy 1 condition (higher-intensity therapist training and lower-intensity leadership training) had a lower risk of nonresponse and drop-out than patients in the other implementation strategy conditions where the therapists received less intensive training. Those in the implementation strategy 2 condition, where both the therapists and the leaders received lower-intensity training, were more likely to not respond to the treatment and more likely to drop out than in all other conditions. Based on fidelity checks of 11,491 treatment sessions, nearly 90 percent of the sessions across all four implementation conditions were conducted with fidelity. Fidelity above 80 percent is perceived reasonable high [10], hence adherence does not seem to explain the differences in nonresponse and drop-out across the implementation conditions. Yet, the data did not allow for direct comparison of fidelity and PTSS outcomes. A likely explanation of the seemingly larger positive treatment effect of implementation strategy 1 is that more thorough case consultations based on audio recordings enhances the possibility of individual tailoring of the consultations for the individual patient. Also, by providing case consultations based on "real time" data, the therapist might get help, advice, and support towards a more proactive approach towards potential barriers to and in therapy. A recent study reported that therapists' self-reports of higher competency with TF-CBT predicted positive treatment response [22]. This might help us understand why implementation strategy 1 with higher-intensity therapist training predicted positive treatment responses among the patients because it may have helped the therapists feel competent and encouraged them to stay with the important exposure work. This is an important finding that should be considered in future studies to generate knowledge on building therapist competency. For example, future studies could consider investigating whether high-intensity therapist training might strengthen general therapeutic skills beyond the specific EBP skills in focus.

Training in implementation leadership is related to a more positive therapist view on implementation leadership and climate (e.g., [1, 53]), however less knowledge is available related to the association between leadership training and patient outcomes. From the results, it seems like leadership support might positively affect patient nonresponse and drop-out, although not enough to compensate for less intensive therapist training. A recent qualitative study [9] including first-level leaders (*n* = 11) from some of the same clinics that took part in the current study demonstrated that training in implementation leadership through the LOCI strategy made the leaders realize and act upon their role as implementation leaders in line with identified key dimensions of implementation leadership where the leader is “knowledgeable, supportive, perservant, available, encouraging, and effectively communicate about the importance of the EBP” [2]. In turn this might have influenced therapist behaviour and patient outcomes [21]. Sustainability of EBPs is important from a system level, and as such, strengthening of leaders and systems have huge potentials. A systematic review found a positive relationship between leadership and patient outcomes [64], but there is a need for more in-depth knowledge on if and in what way implementation leadership training is associated with implementation and patient outcomes. Future studies should include economic evaluations of the costs and benefits of high-intensity leadership training versus high-intensity therapist training. Even if the latter was associated with more favorable patient outcomes in the current study, we need understanding about socioeconomic impacts to make evidence-informed decisions and priorities.

Finally, during the Covid-19 pandemic, both therapist and leader training and many treatment sessions were entirely digital. It is optimistic that this approach did not seem to have any substantial impact on nonresponse or drop-out. As the society may move towards more digital solutions including tele-mental health following the Covid-19 pandemic, more research is needed to understand how digital training and treatment best can be utilized to benefit both therapists and patients. In addition, there is a need for evidence-based tools to determine which children that could benefit from tele-mental health [49]. Feedback from the therapists in the current implementation effort suggest that it might be important to have at least one face-to-face therapy session to build trust and alliance before moving into the virtual therapy room. This is an empirical question which should be tested so that we can make evidence-based recommendation for digital therapy for PTSD among youth. Based on previous research, we know that the first treatment session(s) is vital to reduce drop-out through motivating caregiver attendance and approval [44], addressing caregiver avoidance, and build child-therapist alliance [66]. This is in line with the findings that there were most drop-outs during the first two phases (40% from the Skills phase and 39% from the Narrative phase, whereas 11 percent dropped out from the Consolidation phase. Similarly, a study with 8482 adults from 24 countries reported that dropout from mental health treatment was more prevalent early in the treatment, particularly after the second visit [62]. We cannot know from the current data whether early termination is due to the patients having reached the desired effect or whether the treatment is considered too challenging or non-useful. As it is challenging to predict on an individual level what leads to treatment interruptions, therapists should address drop-out and talk with the child and family about the treatment components, expectations, how the therapy can be adapted to the individual child, and what treatment interruptions may entail. As higher pretreatment symptom load was associated with increased risks of continuing high symptom level following treatment, therapists should screen for trauma exposure and related post-traumatic symptoms and monitor these throughout the therapy process to include this knowledge into the treatment planning. It is also important to identify those who experienced symptom worsening from before to after treatment, which should receive focus in future studies. As such, the results, both from this and previous studies, emphasize the importance of personalized treatment and knowledge of the patient you meet – both to ensure good therapy and to avoid drop-out.

On a system level, we must ensure that evidence-based therapy is well implemented in the services. By moving the focus to therapy and implementation factors while simultaneously focusing on individual care, we might increase effective therapy. According to the meta-analytic study by de Haan et al. [18], therapist and treatment factors are stronger predictors of drop-out than child and family factors, which is partly supported in the current study. Furthermore, a meta-analysis including more than 32 000 youth showed that demographic and pre-trauma factors are only weakly correlated to the development of PTSD, whereas post-trauma factors and certain PTSS are more strongly related to the development of PTSD [56]. This might indicate that clinicians and leaders, as well as future studies on nonresponse and drop-out, should be attentive towards both pre- and post-treatment factors as well as post-trauma factors. For example, therapists should address drop-out in one of the first sessions to prevent drop-out, which is most likely to happen during the first two treatment phases. Lastly, leaders should receive the support needed to be effective implementation leaders.

By using a relatively large sample, the present study investigated both individual, therapist, and implementation factors likely to influence nonresponse and drop-out from TF-CBT. The need for research on discrete, multi-faceted, and tailored implementation strategies has been underscored in the implementation literature [47], and this study expands the previously suggested barriers-to-treatment models related to drop-out at the individual, setting, community, and culture levels [40] by demonstrating the importance of implementation strategies for successful treatment outcomes. The results from this study can be used to make informed decisions on implementation strategies, and be helpful for understanding how to best organize training and implement evidence-based practices in an efficient way. The study was conducted in a real-world clinical context, which indicates that the results might mirror actual practice. Nevertheless, the naturalistic design also poses some limitations to the study. We had less control of the data collection, so we cannot rule out reporting bias, e.g., towards high-responding patients. Furthermore, data collection was based on the strict minimal principal, which was deemed ethical as this study was conducted as part of regular practice, yet it means that we lack information that would have strengthen the study. We are unfamiliar with potential barriers related to the therapist's [44] or the patient's [27] experience of the therapeutic relationship, which in previous research has been demonstrated to predict drop-out. As in most literature on PTSD treatment [37], we had only minimal demographic information about the participants. For example, we did not have information on ethnic minority background, which in previous research has shown a higher risk of treatment drop-out [18]. There was an overlap of clinics in the different implementation conditions which might pose a limitation when interpreting the findings. Yet, as some therapists who received training within the implementation strategy 1 condition continued to work with TF-CBT in the implementation strategy 2 condition, it might indicate that the results are robust. Yet, the effect size estimates were relatively low for all variables, which should be noted to avoid possibly overvaluing the observed effects [24].

The lack of clear definitions and operationalizations of nonresponse (e.g., treatment response, remission, recovery, treatment nonresponse, and worsening) [57] and drop-out hampers generalization across studies and may lead to inconsistent results. A need-based definition for drop-out has been suggested, where "the optimal number of treatment sessions a client requires should vary based on their need at intake" [20]. The current study used therapist judgement of treatment drop-out, and even if the therapists were trained to make judgement of number of treatment session based on the patients’ individual needs, we did not have information on the actual choices being made, hence there was probably natural variations among therapists on how they interpreted and recorded drop-out. Future studies should investigate whether a more systematic approach toward recording the optimal number of treatment sessions a client requires based on their need at intake might mitigate the disadvantages of previous definitions related to therapist judgement (e.g., variations between therapists) or number of treatment sessions (assumption that all patients need the same number of sessions). In this respect, future studies should consider a consensus-driven approach as suggested by Varker and colleagues [57]. Future studies should extend beyond a drop-out/non-drop-out dichotomy by including the information at which point a dropout occurred. Most importantly, the complexity of individual and implementation factors on treatment response should be considered to build the evidence base needed for children and adolescents to respond optimally to TF-CBT and other evidence-based treatment methods for mental health disorders. The goal is that more children should get the best treatment possible.

Lastly, we did not have data on PTSS at the time of drop out, which means that we do not know whether the patients dropped out because they had already experienced adequate improvement or because they did not experience any improvement. Hunsley and colleagues [28], among others, argue that the effect of the treatment affects motivation and that some patients might discontinue therapy if they experience no improvement from the treatment (nonresponders) or if expected progress already has been reached (early responders). One study found that adolescents who drop out from depression treatment have comparable clinical outcomes to those who do not drop out [43], signifying that future studies should include clinical outcomes measures at the time of patient drop-out. It has been suggested that one way to prevent drop-out could be to implement more intensive treatment programs with several sessions within a shorter period, with existing data showing a 90–100 percent retention rate [58]. Future studies on PTSD treatment for children and adolescents should investigate this hypothesis, which would potentially have benefits both on the individual and societal level. As previous research suggests that children who drop out from treatment show greater impairment both at home, at school, and in the community [32], we also encourage future studies to investigate whether there is an association between psychosocial functioning and nonresponse and drop-out.

## Data Availability

The datasets generated and analysed during the current study are not publicly available as we do not have consent from the participants to share the data, but are available from the corresponding author on reasonable request.
